# Chronic Inflammatory Microenvironment in Epidermodysplasia Verruciformis Skin Lesions: Role of the Synergism Between HPV8 E2 and C/EBPβ to Induce Pro-Inflammatory S100A8/A9 Proteins

**DOI:** 10.3389/fmicb.2018.00392

**Published:** 2018-03-07

**Authors:** Marta Podgórska, Monika Ołdak, Anna Marthaler, Alina Fingerle, Barbara Walch-Rückheim, Stefan Lohse, Cornelia S. L. Müller, Thomas Vogt, Mart Ustav, Artur Wnorowski, Magdalena Malejczyk, Sławomir Majewski, Sigrun Smola

**Affiliations:** ^1^Institute of Virology, Saarland University Medical Center, Homburg, Germany; ^2^Department of Histology and Embryology, Center of Biostructure Research, Medical University of Warsaw, Warsaw, Poland; ^3^Department of Dermatology, Saarland University Medical Center, Homburg, Germany; ^4^Icosagen Cell Factory OÜ, Institute of Technology, University of Tartu, Tartu, Estonia; ^5^Diagnostic Laboratory of STDs, Department of Dermatology and Venereology, Medical University of Warsaw, Warsaw, Poland; ^6^Department of Dermatology and Venereology, Medical University of Warsaw, Warsaw, Poland

**Keywords:** HPV, E2, epidermodysplasia verruciformis, inflammation, C/EBP, S100A8/A9

## Abstract

Persistent genus β-HPV (human papillomavirus) infection is a major co-factor for non-melanoma skin cancer in patients suffering from the inherited skin disease epidermodysplasia verruciformis (EV). Malignant EV lesions are particularly associated with HPV type 5 or 8. There is clinical and molecular evidence that HPV8 actively suppresses epithelial immunosurveillance by interfering with the recruitment of Langerhans cells, which may favor viral persistence. Mechanisms how persistent HPV8 infection promotes the carcinogenic process are, however, less well understood. In various tumor types chronic inflammation has a central role in tumor progression. The calprotectin complex consisting of S100A8 and S100A9 proteins has recently been identified as key driver of chronic and tumor promoting inflammation in skin carcinogenesis. It induces chemotaxis of neutrophil granulocytes and modulates inflammatory as well as immune responses. In this study, we demonstrate that skin lesions of EV-patients are massively infiltrated by inflammatory cells, including CD15^+^ granulocytes. At the same time we observed a very strong expression of S100A8 and S100A9 proteins in lesional keratinocytes, which was mostly confined to the suprabasal layers of the epidermis. Both proteins were hardly detected in non-lesional skin. Further experiments revealed that the HPV8 oncoproteins E6 and E7 were not involved in S100A8/A9 up-regulation. They rather suppressed differentiation-induced S100A8/A9 expression. In contrast, the viral transcription factor E2 strongly enhanced PMA-mediated S100A8/A9 up-regulation in primary human keratinocytes. Similarly, a tremendous up-regulation of both S100 proteins was observed, when minute amounts of the PMA-inducible CCAAT/enhancer binding protein β (C/EBPβ), which is expressed at low levels in the suprabasal layers of the epidermis, were co-expressed together with HPV8 E2. This confirmed our previous observation that C/EBPβ interacts and functionally synergizes with the HPV8 E2 protein in differentiation-dependent gene expression. Potent synergistic up-regulation of S100A8/A9 was seen at transcriptional and protein levels. S100A8/A9 containing supernatants from keratinocytes co-expressing HPV8 E2 and C/EBPβ significantly induced chemotaxis of granulocytes in migration assays supporting the relevance of our finding. In conclusion, our data suggest that the HPV8 E2 protein actively contributes to the recruitment of myeloid cells into EV skin lesions, which may support chronic inflammation and progression to skin cancer.

## Introduction

Human papillomaviruses (HPVs) infect keratinocytes of mucosa and skin and cause a wide range of clinical outcomes from benign to malignant lesions. Mucosal HPV types have a well-established role in anogenital tumors, particularly in cervical carcinoma. Cutaneous HPVs of the genus beta (β-HPV) were first discovered in patients with epidermodysplasia verruciformis (EV) (Majewski and Jablonska, 2006). This rare genetic skin condition mostly results from recessive mutations in *EVER1* or *EVER2* genes, which confer predisposition to persistent β-HPV infection (Shamanin et al., 1994; Ramoz et al., 2002). EV begins early in childhood with widespread flat warts and macules, which can, later in life, evolve to cutaneous squamous cell carcinoma (SCC), mainly at sun-exposed sites. Multiple HPVs, e.g., HPV5, 8, 17, 20, 27, 38, or 47, can be found within the EV skin lesions but HPV5 or 8 are the most frequently detected in EV-associated skin carcinomas (Orth et al., 1978; Orth, 1986; Pfister, 2003).

Viral persistence is a major prerequisite for HPV-induced tumor development in cervix uteri and potentially in the skin and it requires an escape of the virus from host immune surveillance (zur Hausen, 2009; Smola, 2017; Smola et al., 2017). Recently, we have proposed a novel molecular mechanism how HPV8 might disrupt innate immunity in skin. We found reduced numbers of antigen-presenting Langerhans cells in EV lesions and showed that it is a consequence of HPV8-mediated suppression of CCL20, which is a chemokine attracting Langerhans cells (Sperling et al., 2012; Smola, 2014).

A set of *in vivo* data employing transgenic mouse models established a role of EV-associated HPVs in the development of skin tumors. HPV38, 20, and 27 transgenic mice expressing E6 and E7, the main viral oncoproteins, displayed increased susceptibility to carcinogenesis after UV or chemical treatment (Michel et al., 2006; Viarisio et al., 2011). In mice expressing the complete early region of HPV8 under the keratin 14 (K14) promoter even spontaneous development of skin cancers was observed. Transcript analysis in the affected tissue revealed high mRNA levels of the viral transcription factor E2, which prevailed over E7 and E6 and did not change during tumor progression (Schaper et al., 2005). Interestingly tumor development was also observed in transgenic mice expressing only the HPV8 E2 gene under the K14 promoter and this process was accompanied by a massive stromal infiltration of immune cells (Pfefferle et al., 2008). These findings emphasized the importance of the E2 protein in tumor formation and raised the hypothesis that E2 might augment tumor-promoting inflammatory responses in skin.

Recently, S100A8 (calgranulin A, MRP-8) and S100A9 (calgranulin B, MRP-14) have been identified as key drivers of skin carcinogenesis in a mouse model (Gebhardt et al., 2008). Both proteins belong to the S100 multigenic family of non-ubiquitous Ca^2+^-binding low molecular weight proteins. They associate in a higher-ordered heteromeric complex called calprotectin, which is essential for their biological activity (Leukert et al., 2006). In skin their expression has been observed under inflammatory conditions, such as wound healing or psoriasis (Kerkhoff et al., 2012). Beside the intracellular activity of S100A8 and S100A9 proteins, they are also found in the extracellular milieu, where they function as damage-associated molecular pattern molecules (DAMPs) or alarmins and induce immune cell migration, particularly of granulocytes (Ryckman et al., 2003).

In this report, we demonstrate that S100A8/A9 proteins are potently up-regulated in HPV8-positive EV lesions and S100 expression was paralleled by a strong inflammatory response. Detailed molecular analysis revealed that not the HPV8 E6 or E7 oncoproteins, but the viral transcription factor E2 plays a major role in S100A8/A9 induction.

## Materials and Methods

### Ethics Statement

This study was carried out in accordance with the recommendations of the Declaration of Helsinki with written informed consent from all subjects. All subjects gave written informed consent in accordance with the Declaration of Helsinki. The protocol was approved by the Bioethics Committee at the Medical University of Warsaw, Poland, and the Saarland University at the Saarland Ärztekammer.

### Immunohistochemistry, Immunofluorescence, and HPV Genotyping

Formalin fixed paraffin-embedded (FFPE) skin specimens from EV-lesions were obtained from the Department of Dermatology, Medical University of Warsaw, Warsaw, Poland. The presence of HPV8 was confirmed by quantitative real-time PCR (qRT-PCR) as described in Weissenborn et al. (2010). Sections were stained with mouse monoclonal anti-CD15 antibody (clone Carb-3, Dako, Glostrup, Denmark), rabbit monoclonal anti-S100A8 antibody (clone EPR3554; Novus Biologicals, Cambridge, United Kingdom), rabbit polyclonal anti-S100A9 antibody (H-90, sc-20173, Santa Cruz Biotechnology, Heidelberg, Germany) or mouse anti-CD45 (Abcam, Cambridge, United Kingdom). Staining was performed using the Dako instrument Autostainer Plus (Dako) or Immpress AP Reagent kit (Vector, Burlingame, CA, United States). For immunofluorescence, cells were grown on cover slips, fixed with 2% buffered paraformaldehyde, permeabilized with 0.1% Triton X-100 and stained with rabbit anti-HPV8 E2 (from Mart Ustav, University of Tartu, Tartu, Estonia) and mouse monoclonal anti-pan-cytokeratin (clone C11; Sigma-Aldrich, Steinheim, Germany) and secondary goat anti-rabbit Alexa Fluor 546 goat and goat anti-mouse Alexa Fluor 488 (Life Technologies, Eugene, OR, United States), respectively.

### Plasmid Constructs

Expression vectors of HPV8 E2 and E6/E7 cloned in pLXSN, HPV8 E2, E6, E7, HPV16 E2, C/EBPβ, cloned in pcDNA3.1+ and HPV8 E2ΔC in pXJ42 were reported previously (Hadaschik et al., 2003). S100A8 in pcDNA3.1/myc-His(-) and S100A9 in pcDNA3.1+ expression vectors were kind gifts from Peter Angel (German Cancer Research Centre, Heidelberg, Germany). The S100A8 luciferase reporter construct containing a fragment (-917 to +456 bp) of the murine S100A8 promoter region cloned upstream of the firefly luciferase gene in the pGL2-basic vector was a kind gift from Kenneth Hsu (Sydney, NSW, Australia). It comprises a functional C/EBPβ binding site, which is highly conserved in mammalian S100A8 promoters, particularly in human and murine promoters, and has been shown to bind to C/EBPβ by chromatin immunoprecipitation (Miao et al., 2012).

### Cell Culture and Retroviral Infection

Normal human foreskin keratinocytes (NFKs) were cultured in KBM-Gold medium with supplements (Lonza, Basel, Switzerland). NFK stably expressing HPV8 E2 or E6/E7 were generated by retroviral gene transfer as previously described (Oldak et al., 2010). Viral gene expression was confirmed by HPV8 E2-, E6-, and E7-specific qRT-PCR. The HPV-negative skin SCC-derived RTS3b cell line (Purdie et al., 1993) was grown as previously reported (Hadaschik et al., 2003). Organotypic 3-dimensional cultures (OC) were generated as described in Smola et al. (1993) with minor modifications. Dermal equivalents were prepared by seeding 0.5 × 10^6^ primary foreskin fibroblasts (usually between passage 3 and 6) in 4 mg/ml rat-tail collagen. Next day 0.5 × 10^6^ RTS3b cells were seeded onto the fibroblast-collagen matrix. Twenty-four hours later OC were lifted on metal grids at the air-medium interphase, grown for 14 days, fixed with 4% buffered paraformaldehyde, embedded in paraffin and sectioned.

### Granulocyte Isolation and Migration Assay

Granulocytes were obtained from whole blood of healthy volunteers by dextran-sedimentation of red blood cell pellets. The purity of granulocyte isolation was 93–99% as determined by CD15 and CD66 surface expression (mouse anti-human CD15-PE BD Biosciences, Heidelberg, Germany and mouse anti-human CD66abce-APC, Miltenyi Biotec, Bergisch Gladbach, Germany). Contaminating monocytes, T- and B-cells in granulocyte isolates were discriminated with CD14-FITC, CD3-FITC, and CD19-PE antibodies. Prior to the assessment of migration, granulocytes were cultured for 2 h under endotoxin-free conditions in RPMI 1640 supplemented with 5% FCS, 1% penicillin-streptomycin and 1% sodium pyruvate. 0.25 × 10^6^ granulocytes were then seeded onto transwell chambers placed in 24-well plates (3 μm pore size, Corning Costar Corp., Corning, NY, United States). The migration capacity of granulocytes was assessed after 2 h. Background migration in culture medium only was subtracted.

### Transient Transfection and Luciferase Assay

Normal human foreskin keratinocytes were transfected with TransFast reaching transfection efficiencies between 10 and 15% (Promega, Mannheim, Germany) and RTS3b cells with Lipofectamine 2000 reagent reaching transfection efficiencies between 50 and 70% (Invitrogen, Karlsruhe, Germany) according to the manufacturers’ protocols. Twenty-four hours later NFKs were stimulated with PMA for another 24 h (Sigma-Aldrich, Taufkirchen, Germany). Afterward, the cells were lysed and assayed for luciferase activity as described previously (Marthaler et al., 2017). The values of luciferase activities were normalized to protein content of the respective lysates.

### RNA Isolation and qRT-PCR

RNA was isolated using NucleoSpin RNA kit (Macherey-Nagel, Düren, Germany) and cDNA was generated from 0.5 to 1 μg of RNA with the Maxima Reverse Transcriptase (Thermo Fisher Scientific, Rockford, IL, United States). qRT-PCR was performed using gene-specific primers and the Universal Probe Library System (Roche, Mannheim, Germany) as follows: S100A8: 5′-CAAGTCCGTGGGCATCAT-3′, 5′-GACGTCGATGATAGAGTTCAAGG-3′, probe #78, S100A9: 5′-GTGCGAAAAGATCTGCAAAA-3′, 5′-CCAGCTGCTTGTCTGCATTT-3′, probe #85, RPL13A: 5′-AGCGGATGAACACCAACC-3′, 5′-TTTGTGGGGCAGCATACTC-3′, probe #28, HPV8 E2: 5′-GACGGCGATCAACCTCAA-3′, 5′-CTCCCCTTTGTGACCGTTT-3′, probe #22, HPV8 E6:5′-CCGCAACGTTTGAATTTAATG-3′, 5′-ATTGAACGTCCTGTAGCTAATTCA-3′, probe #13 and HPV8 E7: 5′-AGGAATTACCAAACGAACAGGA-3′, 5′-CACGGTGCAACAATTTTGAATA-3′, probe #63. Expression levels were measured using LightCycler 480 II instrument (Roche) and normalized to RPL13A housekeeping gene expression (Marthaler et al., 2017).

### Western Blot Analysis and ELISAs

The cells were lysed in buffer containing 62.5 mM Tris-Cl pH 6.8, 10% glycerol and 2% SDS. Thirty micrograms of the samples were separated by 9.5–20% gradient SDS-PAGE and subjected to Western blotting. Anti-S100A8, anti-S100A9, mouse monoclonal anti-actin (clone AC-15, Sigma-Aldrich, St. Louis, MO, United States) as well as horseradish peroxidase-labeled goat anti-rabbit and rabbit anti-mouse (both Sigma-Aldrich) antibodies were used followed by chemiluminescence detection (Thermo Fisher Scientific). ChemiDoc XRS^+^ Molecular Imager and Quantity One analysis software (both Bio-Rad, Philadelphia, PA, United States) were used for quantification. Interleukin-8 (IL-8, CXCL8), Epithelial-derived Neutrophil-Activating peptide 78 (ENA-78, CXCL5), Neutrophil Activating Protein-2 (NAP-2, CXCL7) and Growth-Regulated Oncogene-α (GRO-α, CXCL1) concentrations were measured with DuoSet (R&D Systems, South Beloit, IL, United States) according to the manufacturer’s protocols.

### Statistical Analysis

Statistical differences were determined with unpaired *t*-test using Prism 5 software (GraphPad Software, La Jolla, CA, United States). Significances are indicated by asterisks (^∗^*p* < 0.05, ^∗∗^*p* < 0.01, ^∗∗∗^*p* < 0.001, ^∗∗∗∗^*p* < 0.0001).

## Results

**FIGURE 1:**
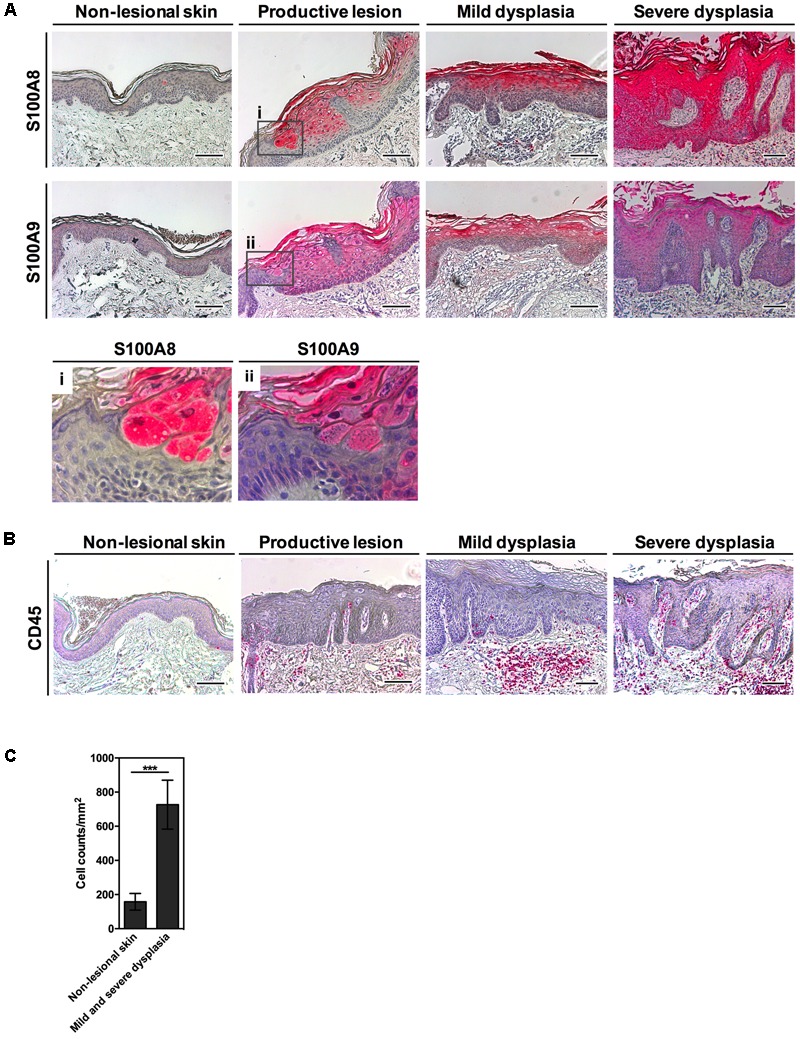
Strong expression of S100A8/A9 in HPV8-positive EV-lesions corresponds to an inflammatory infiltrate. **(A)** Serial sections of HPV8-positive EV-lesions were analyzed by IHC for S100A8 and S100A9 (magnifications in **i** and **ii**) or **(B)** CD45 (all red) and counterstained with hematoxylin. Scale bars 100 μm. **(C)** Infiltrating immune cells were counted in the stroma of mild and severe dysplasia of EV-lesions versus non-lesional skin of EV-patients based on hematoxylin counterstaining and are given as cell counts per mm^2^. Values represent counts ± SD from six different areas. ^∗∗∗^*p* < 0.001, unpaired *t*-test.

In this study, we investigated S100A8 and S100A9 protein expression in the skin of EV-patients. In non-lesional skin both proteins were occasionally detectable in the keratinocytes of the granular layer or were completely lacking (**Figure 1A**). HPV8-positive lesional skin of EV-patients, however, revealed a dramatic induction of S100A8 and S100A9 proteins in keratinocytes of suprabasal spinous and granular layers. In productive EV-lesions staining of both proteins was particularly prominent in keratinocytes displaying viral cytopathic effects as demonstrated in higher magnifications (**Figures 1A i** and **ii**). Also in mild and severe dysplasia S100A8 and S100A9 staining was strongly enhanced and mostly confined to the suprabasal layers of infected epidermis. Notably, S100A8 and S100A9 expression in lesional epidermis was paralleled by significant stromal infiltration with immune cells as shown by CD45 staining, a pan-marker for leukocytes, and quantification of stromal infiltrating cells (**Figures 1B,C**).

**FIGURE 2:**
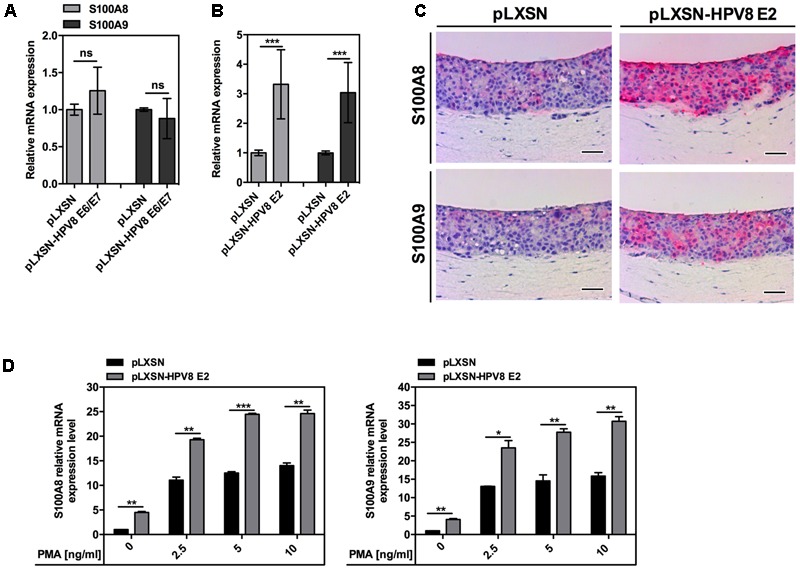
Not HPV8 E6/E7 oncoproteins but the E2 transcription factor induces both S100A8 and S100A9. S100A8/A9 mRNA levels in NFK stably expressing **(A)** HPV8 E6/E7 or **(B)** HPV8 E2 and corresponding control pLXSN cells were measured by qRT-PCR in relation to RPL13A. The amount of S100A8 and S100A9 in pLXSN control cells was set at 1. Shown are the mean values ± SD from *n* ≥ 2 independent experiments performed in duplicates. **(C)** Serial sections of OC of HPV8 E2-expressing or control pLXSN RTS3b cells were analyzed by IHC for S100A8 and S100A9 (both red) and counterstained with hematoxylin. Scale bars 100 μm. **(D)** NFK stably expressing HPV8 E2 or control pLXSN cells were stimulated with PMA or DMSO as a vehicle control and 24 h later S100A8 and S100A9 mRNA levels were measured by qRT-PCR in relation to RPL13A. Shown are the mean values ± SD from one representative experiment out of three performed in duplicates. ns, not significant, ^∗^*p* < 0.05, ^∗∗^*p* < 0.01, ^∗∗∗^*p* < 0.001, unpaired *t*-test.

To investigate the potential impact of HPV8 early proteins on S100A8 and S100A9 induction, normal human keratinocytes (NFKs) and the RTS3b skin keratinocyte cell line were engineered to stably express HPV8 E2 or combined E6/E7 genes by retroviral gene transfer. Expression of HPV8 E2, E6, and E7 genes was confirmed by qRT-PCR and E2 additionally on protein level by double immunofluorescence (Supplementary Figure S1). Presence of HPV8 E6/E7 in NFK did not influence S100A8 and S100A9 expression (**Figure 2A**). However, a significant increase in S100A8/A9 mRNA levels was observed in HPV8 E2-expressing NFK (**Figure 2B**). This was further validated at the protein level by strong S100A8 and S100A9 IHC staining in OC of HPV8 E2-expressing RTS3b keratinocytes compared to control cells with empty pLXSN vector only (**Figure 2C**).

Since in EV-lesions S100A8 and S100A9 staining was confined to the suprabasal more differentiated layers, we were interested whether HPV8 E2 might cooperate with differentiation-regulating factors to up-regulate the calgranulins. To test this hypothesis, HPV8 E2-expressing NFKs were stimulated with phorbol 12-myristate 13-acetate (PMA), a well-known activator of protein kinase C (PKC) and inducer of keratinocyte differentiation (Agarwal et al., 1999; Balasubramanian et al., 2000; Goethe et al., 2007). PMA significantly enhanced the effect of HPV8 E2 on S100A8 and S100A9 mRNA in NFK from two different donors (**Figure 2D** and Supplementary Figure S2).

**FIGURE 3:**
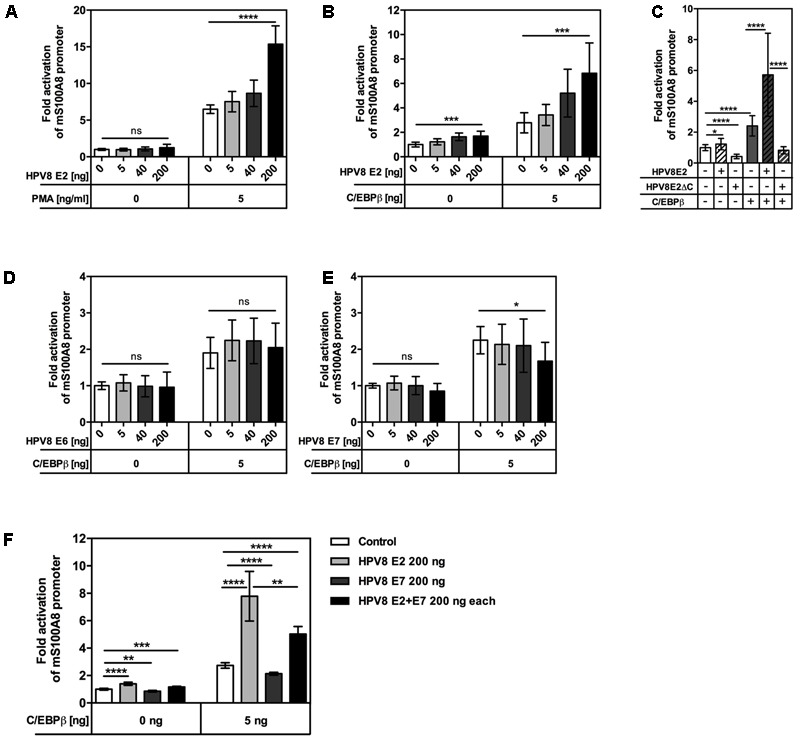
HPV8 E2 enhances PMA- or C/EBPβ-induced activation of the S100A8 promoter in human keratinocytes. **(A)** 0.1 × 10^6^ NFKs were seeded onto 12-well plates, next day co-transfected with 0.2 μg S100A8 reporter vector and 0.005, 0.04, or 0.2 μg HPV8 E2 expression vector and 6 h later stimulated with PMA or DMSO as a vehicle control. 0.61 × 10^5^ RTS3b cells were seeded onto 12-well plates and next day co-transfected with 0.2 μg S100A8 reporter vector together with 0.005 μg C/EBPβ and in **(B)** 0.005, 0.04, or 0.2 μg HPV8 E2, **(C)** 0.2 μg HPV8 E2 or HPV8 E2ΔC, **(D)** 0.005, 0.04, or 0.2 μg HPV8 E6, **(E)** 0.005, 0.04, or 0.2 μg HPV8 E7 or **(F)** HPV8 E2 and E7 expression vectors (0.2 μg each). Total amount of DNA in all transfections was adjusted up to 0.8 μg with pcDNA3.1+ empty vector. 24 h post-transfection the luciferase activity was measured and normalized to protein concentration. Control transfection was set at 1. Shown are the mean values ± SD from *n* ≥ 3 independent experiments performed in triplicates. ns, not significant, ^∗^*p* < 0.05, ^∗∗^*p* < 0.01, ^∗∗∗^*p* < 0.001, ^∗∗∗∗^*p* < 0.0001, unpaired *t*-test.

To study whether regulation took place at the promoter level, the interplay of PMA activation and HPV8 E2 expression was examined in reporter gene assays. In NFK, transient expression of HPV8 E2 alone had only a minor effect, while PMA stimulation strongly synergized with HPV8 E2 in S100A8 promoter activation (**Figure 3A**). Others and we have previously demonstrated that PMA potently induces the transcription factor C/EBPβ in primary human keratinocytes (Park et al., 2008; Sperling et al., 2012). Furthermore, our previous data had shown that HPV8 E2 can bind to and synergize with C/EBPβ to transactivate the involucrin promoter (Hadaschik et al., 2003). Notably, a functional C/EBPβ binding site is also highly conserved in mammalian S100A8 promoters, particularly in human and murine promoters (Miao et al., 2012). Since C/EBPβ is expressed at low levels in suprabasal layers of the skin (Sperling et al., 2012), minute amounts of C/EBPβ (5 ng) were co-transfected together with the HPV8 E2 expression vector in RTS3b cells. In these cells transient transfection of HPV8 E2 alone significantly activated the murine S100A8 promoter up to 1.64-fold while co-expression of HPV8 E2 and C/EBPβ significantly increased promoter activity in a dose-dependent manner up to 7-fold, similar to the results obtained with the involucrin promoter (**Figure 3B**) (Hadaschik et al., 2003). HPV8 E2 interacts with C/EBPβ via its C-terminus. We therefore investigated the impact of the C-terminal E2 deletion mutant (HPV8 E2ΔC) on S100A8 promoter activity. While full length HPV8 E2 strongly synergized with C/EBPβ, this was not observed for the E2 mutant lacking the C-terminal C/EBPβ-interacting domain (**Figure 3C**). To investigate the influence of HPV8 E6 and E7 on C/EBPβ-induced S100A8 promoter activity, we performed co-transfection experiments in RTS3b cells. Neither E6 nor E7 alone increased C/EBPβ-induced promoter activity. HPV8 E7 even led to a suppression of C/EBPβ-induced promoter activity (**Figures 3D,E**). This corresponded well to our previous observation that HPV8 E7 can interfere with C/EBPβ-mediated induction of the Langerhans cell attracting chemokine CCL20 (Sperling et al., 2012). Since E7 is predominantly expressed in the granular layer of EV-lesions, we investigated its potential impact on the synergism between C/EBPβ and E2 on the S100A8 promoter. HPV8 E7 alone was able to reduce C/EBPβ-dependent promoter activity. When equal amounts of HPV8 E2 and E7 were co-transfected together with C/EBPβ, however, the positive E2 activity prevailed over the negative effect of HPV8 E7 (**Figure 3F**).

**FIGURE 4:**
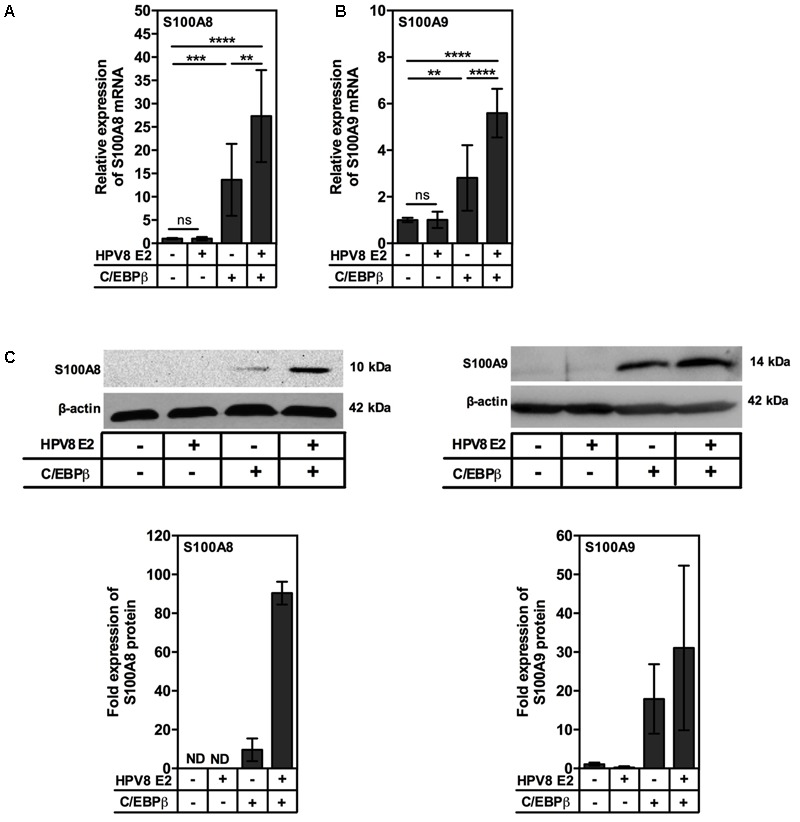
HPV8 E2 synergizes with minute amounts of C/EBPβ to up-regulate S100A8 and S100A9 in human keratinocytes. RTS3b cells were seeded onto 6-cm dishes at a density of 0.4 × 10^6^ cells per dish and transiently co-transfected with 2.64 μg HPV8 E2 and 0.1 μg C/EBPβ expression vectors and 24 h later were analyzed for **(A)** S100A8 and **(B)** S100A9 mRNA expression by qRT-PCR in relation to RPL13A. Shown are the mean values ± SD from *n* = 3 independent experiments performed in duplicates. **(C)** For Western blot analysis 1 × 10^6^ RTS3b cells were seeded onto 10-cm dishes and next day co-transfected with 0.27 μg C/EBPβ and 7.2 μg HPV8 E2 expression vectors. Forty-eight hours later whole-cell extracts were analyzed for S100A8 (left) and S100A9 (right) protein expression. β-actin served as a loading control. Shown is one representative experiment out of *n* = 3. Diagrams summarize *n* = 3 experiments. ND, not detected; ns, not significant, ^∗∗^*p* < 0.01, ^∗∗∗^*p* < 0.001, ^∗∗∗∗^*p* < 0.0001, unpaired *t*-test.

Next, we analyzed the impact of transient HPV8 E2 and C/EBPβ expression on the regulation of endogenous S100A8 and S100A9. Expression of HPV8 E2 led to a strong enhancement of C/EBPβ-mediated S100A8 induction (more than 25-fold, **Figure 4A**) and S100A9 (up to 6-fold, **Figure 4B**) at mRNA level. In parallel experiments the cells were subjected to Western blot analysis. While control cells expressed minimal levels of calgranulins, HPV8 E2 enhanced the impact of C/EBPβ on the levels of endogenous S100A8 and S100A9 proteins up to 90- and 31-fold, respectively, as shown by quantification (**Figure 4C**).

This effect was not seen for its mucosal counterpart HPV16 E2, which did not enhance but rather suppressed C/EBPβ-activated S100A8 promoter activity and S100A8/A9 mRNA levels (Supplementary Figure S3).

**FIGURE 5:**
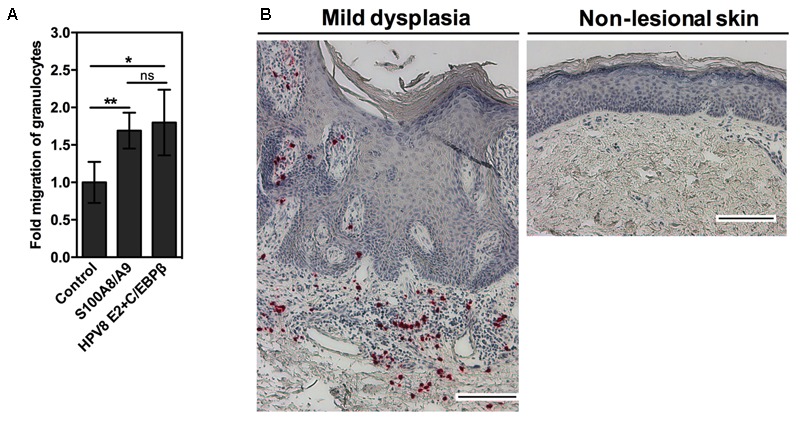
Co-expression of HPV8 E2 and C/EBPβ induces migration of granulocytes *in vitro*. **(A)** Granulocyte migration toward conditioned media collected from RTS3b cells co-transfected with S100A8 and S100A9 or HPV8 E2 and C/EBPβ expression vectors or control vectors was measured by transwell chemotaxis assay. Number of migrated granulocytes toward control conditioned media was set at 1. Shown are the mean values ± SD from *n* = 3 independent experiments performed in duplicates. **(B)** Sections of EV-lesions were stained with anti-CD15 antibody by IHC (red). Counterstaining with hematoxylin reveals the segmented appearance of nuclei in CD15-positive cells indicating infiltration with granulocytes. Scale bars 100 μm. ns, not significant, ^∗^*p* < 0.05, ^∗∗^*p* < 0.01, unpaired *t*-test.

Since the S100A8/A9 complex is chemotactic for neutrophils, we set up an *in vitro* migration assay. Freshly prepared conditioned media from transiently transfected keratinocytes were applied onto transwells containing granulocytes. Keratinocytes ectopically expressing S100A8/A9 significantly induced the migration of granulocytes. Notably, conditioned media obtained from the keratinocytes co-transfected with HPV8 E2 and minute amounts of C/EBPβ induced migration of granulocytes at least as strong as overexpressed S100A8/A9 (**Figure 5A**).

To validate this finding *in vivo*, the EV-lesions were further investigated. We observed that the stroma of HPV8-positive EV-lesions was strongly infiltrated with CD15-positive granulocytes in comparison to non-lesional skin, where granulocytes were not detected (**Figure 5B**).

## Discussion

**FIGURE 6:**
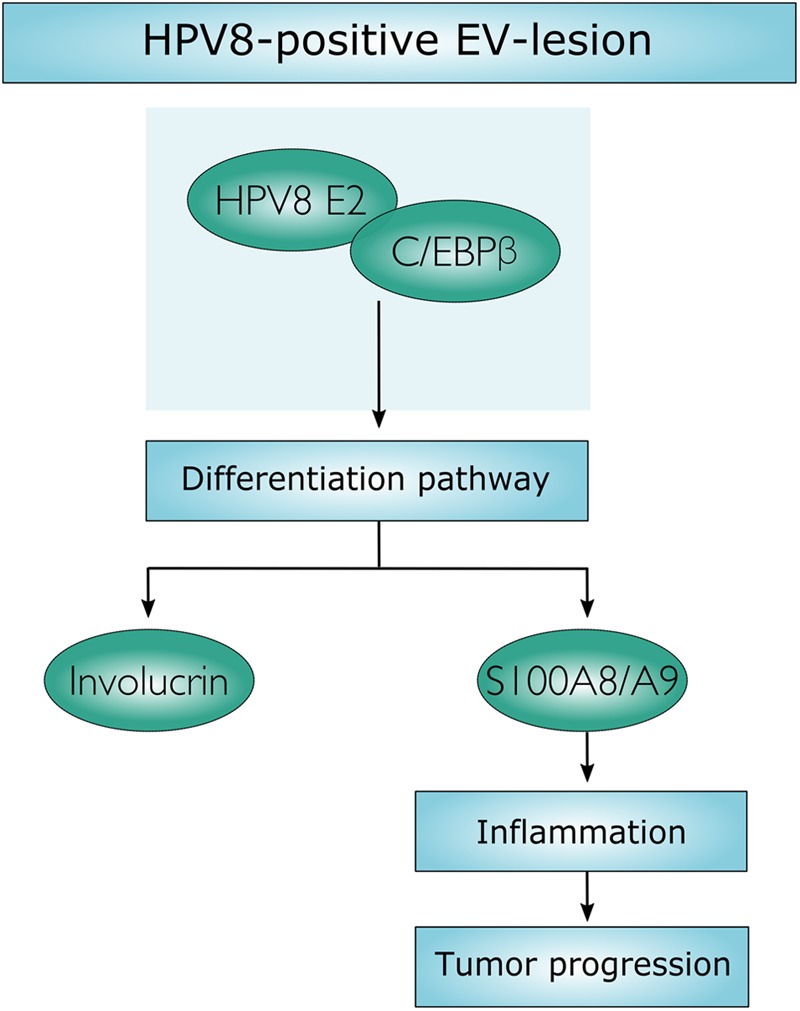
Schematic presentation. Molecular mechanism, by which HPV8 E2 synergizes with C/EBPβ to induce keratinocyte differentiation and to up-regulate S100A8/A9 expression.

In this study, we demonstrate that HPV8-positive skin lesions of EV patients are characterized by a dramatic increase of suprabasal alarmin S100A8/A9 expression and stromal inflammation starting in productive lesions and still observed in mild and severe dysplasia. Analysis of the underlying molecular mechanism revealed that the viral transcription factor HPV8 E2 synergizes with the cellular differentiation-dependent transcription factor C/EBPβ to up-regulate S100A8/A9 expression and to enhance recruitment of granulocytes. Our data indicate that the HPV8 E2 protein hijacks a differentiation-associated pathway to create a chronic inflammatory microenvironment in EV-patients that may pave the way for carcinogenic progression (**Figure 6**).

Epidermodysplasia verruciformis patients are highly susceptible to β-HPV-associated skin carcinogenesis at sun-exposed sites (Smola, 2014). However, the HPV-mediated molecular mechanisms that support the carcinogenic process are poorly understood so far. Our study strongly indicates that the cutaneous β-HPV type 8 can actively trigger inflammation, a hallmark of cancer (Hanahan and Weinberg, 2011). In human organ transplant recipients as well as in mouse models of skin carcinogenesis, this involves the induction of the alarmins S100A8/A9 promoting a pro-tumorigenic inflammatory microenvironment (Gebhardt et al., 2008; Iotzova-Weiss et al., 2015). S100A8/A9 proteins gained a unique interest over the last decades with respect to their role in cancer progression since they can propel cancer cell proliferation, migration, and invasion (Bresnick et al., 2015). Despite the well-defined role of S100A8/A9 in carcinogenesis, however, little is known about factors altering their expression, particularly in HPV-associated carcinogenesis.

Our data clearly demonstrate that S100A8/A9 expression is accompanied by strong immune cell infiltration starting during the productive phase of viral infection in EV-lesions. Tumor-promoting inflammation was previously also observed in mucosal HPV-driven carcinogenesis (Schroer et al., 2011; Walch-Ruckheim et al., 2015, 2016; Smola, 2017; Smola et al., 2017). From published data it was unclear, whether β-HPV oncoproteins contribute to increased inflammatory cytokine production (De Andrea et al., 2007; Akgul et al., 2010). For HPV8 E7, it was rather shown that it interferes with Langerhans recruitment via repression of the chemokine CCL20 (Sperling et al., 2012). This is consistent with observations in anogenital HPV infection, where mucosal high-risk HPV oncoproteins were shown to suppress inflammatory responses in keratinocytes (Guess and McCance, 2005; Smola-Hess and Pfister, 2006; Karim et al., 2011, 2013). Here, we demonstrate that neither expression of the HPV8 E6 nor the E7 protein in primary human keratinocytes resulted in higher levels of S100A8 and S100A9. Rather, our study provides evidence that the β-HPV type 8-encoded transcription factor E2 up-regulates S100A8 and S100A9 on mRNA and protein levels. The relevance of this finding was further supported by the observation that calgranulin expression converges spatially with HPV5 E2 mRNA (Haller et al., 1995) in EV-lesions that display characteristic cytopathic effects of β-HPV infection. Notably, this activity seemed to be a unique feature of cutaneous HPV E2, since mucosal high-risk HPV16 E2 was unable to induce S100A8/A9 mRNA expression.

The HPV E2 protein regulates viral transcription through direct binding via its C-terminal domain to the palindromic ACCN_6_GGT motif in the HPV non-coding regulatory region, which can result either in gene induction or repression (McBride, 2013), and HPV8 E2 was additionally shown to bind to a non-classical site ATCGN_4_CGAT (Akgul et al., 2003). We have previously demonstrated that HPV8 E2 can also modulate the expression of a cellular gene, β4-integrin, via E2-binding sites (Oldak et al., 2004, 2010). E2-expressing cells lose β4-integrin expression, which normally anchors keratinocytes to the basement membrane. This may push E2-expressing cells into suprabasal layers, where they are destined to differentiate. Applying bioinformatic tools, however, we neither detected any classical E2 binding site in the S100A8 and S100A9 promoter regions nor a non-canonical site completely matching with ATCGN_4_CGAT (Akgul et al., 2003). Only in the human S100A8 promoter, a site with some similarities (ATCTGGCTGGAT) was detectable at positions -168 to -156, while not being present in the murine counterpart that was used in this study for reporter assays.

As an alternative mechanism to regulate cellular gene expression, HPV E2 engages cellular transcription factors via direct protein-protein interactions. This was first shown for cellular transcription factors of the C/EBP family (Hadaschik et al., 2003) and also for other factors (McBride et al., 2004; McPhillips et al., 2006; Wu et al., 2016). It was shown that E2 can form a ternary complex with C/EBP factors in a DNA-bound state at a C/EBP-specific binding site within the human involucrin gene, a classical marker of keratinocyte differentiation. As a consequence of its interaction with C/EBP factors, E2 leads to potent activation of the involucrin promoter (Hadaschik et al., 2003). Notably, C/EBPβ also directly binds to the S100A8 promoter region as demonstrated by chromatin immunoprecipitation (Miao et al., 2012). In fact, in our transient transfection experiments in RTS3b cells, HPV8 E2 alone was able to activate the murine S100A8 promoter containing the C/EBP but no putative E2-binding site. Moreover, in both, primary human keratinocytes as well as RTS3b cells, HPV8 E2 significantly enhanced PMA- or C/EBPβ-induced promoter activation, respectively. This strongly supported the notion that the molecular mechanism underlying the synergism between HPV8 E2 and C/EBPβ does not necessarily depend on a classical or non-classical E2-binding site but rather resembles the situation previously described for the human involucrin promoter (Hadaschik et al., 2003). However, with respect to the human S100A8 promoter, we cannot exclude that the ATCTGGCTGGAT sequence might further contribute to the E2 effect. In addition, human and murine S100A8 promoter regions are very similar with respect to their C/EBPβ binding site (Miao et al., 2012) but they are not identical. Therefore, we cannot exclude that the promoter region of the murine gene, we used for the reporter assays, can also bind to other transcription factors than those involved in the regulation of the human gene.

S100A8/A9 are potent pro-inflammatory mediators. Our preliminary analysis indicates that calgranulins are not the only pro-inflammatory factors that are up-regulated in cells co-expressing E2 and C/EBPβ, since we could also detect several other neutrophil attracting chemokines in their supernatants. These included IL-8, ENA-78 and NAP-2. The chemokine GRO-α was expressed constitutively and only slightly but non-significantly up-regulated in the E2 and C/EBPβ expressing cells (Supplementary Figure S4). The exact mechanisms underlying the regulation of these chemokines remain to be determined in the future. In our preliminary analyses, recombinant S100A8/A9 did not lead to induction of these chemokines indicating that their up-regulation was parallel to and not via S100A8/A9. In contrast, it has been demonstrated that calgranulins are involved in the induction matrix-metalloproteinases, such as MMP-9, which are involved in tumor growth and angiogenesis (Moon et al., 2008; Saha et al., 2010; Kerkhoff et al., 2012; Lim et al., 2016). From this it can be speculated that the E2-C/EBPβ-S100A8/A9 axis might exert further pleiotropic effects during carcinogenesis.

Apart from this, S100A8/A9, loss of β4-integrin and the induction of involucrin are all involved in keratinocyte differentiation. A role for HPV8 E2 in keratinocyte differentiation was also supported by observations in transgenic mice expressing the HPV8 E2 protein under the K14-promoter (Pfefferle et al., 2008). Morphologically, their skin presented obvious ulcerations and thinning of the epidermis. This was discussed as a putative consequence of premature keratinocyte differentiation and a reduced pool of proliferative keratinocytes consistent with our previous observation of β4-integrin suppression and involucrin induction (Hadaschik et al., 2003; Oldak et al., 2004, 2010; Pfefferle et al., 2008). Importantly, the skin of HPV8 E2-transgenic mice was also characterized by chronic immune cell infiltration and it was associated with spontaneous tumorigenic progression in a significant proportion of the mice (Pfefferle et al., 2008).

Collectively, these data provided evidence for a role of the E2 protein not only in differentiation but also in inflammation and skin tumor formation. This raised the question, how HPV8 E2 might augment tumor-promoting inflammation in skin.

The data presented in this study may provide this missing link. We demonstrate that HPV8 E2 engages the differentiation-inducing C/EBPβ pathway to up-regulate S100A8/A9 factors, which are not only part of the epidermal differentiation complex (Kuruto-Niwa et al., 1998; Miao et al., 2012) but also part of an inflammatory response promoting tumorigenesis (**Figure 6**).

## Author Contributions

SS, MP, and MO: conceptualization. SS and SL: funding acquisition. SS, MP, MO, AM, SL, and AW: investigation. SS, MP, AM, MO, CM, and BW-R: methodology. SS, CM, TV, MU, MM, and SM: resources. SS: supervision. SS, MP, MO, SL, and BW-R: validation. MP: visualization. SS and MP: writing the original draft. SS, MP, and SL: reviewing and editing the manuscript.

## Conflict of Interest Statement

The authors declare that the research was conducted in the absence of any commercial or financial relationships that could be construed as a potential conflict of interest.
