# Supplementary material for: Chronic Inflammatory Microenvironment in Epidermodysplasia Verruciformis Skin Lesions: Role of the Synergism Between HPV8 E2 and C/EBPβ to Induce Pro-Inflammatory S100A8/A9 Proteins
Source: Front Microbiol. 2018 Mar 7;9:392. doi: 10.3389/fmicb.2018.00392 (PMC5845987; doi:10.3389/fmicb.2018.00392)
Supplement: Supplementary file 1 [file Data_Sheet_1.DOCX]

Supplementary Material

**Chronic inflammatory microenvironment in epidermodysplasia verruciformis skin lesions: role of the synergism between HPV8 E2 and C/EBPβ to induce pro-inflammatory S100A8/A9 proteins**

**Marta Podgórska, Monika Ołdak, Anna Marthaler, Alina Fingerle, Barbara Walch-Rückheim, Stefan Lohse, Cornelia Sigrid Lissi Müller, Thomas Vogt, Mart Ustav, Artur Wnorowski, Magdalena Malejczyk, Sławomir Majewski, Sigrun Smola***

*** Correspondence:** Sigrun Smola: Sigrun.Smola@uks.eu


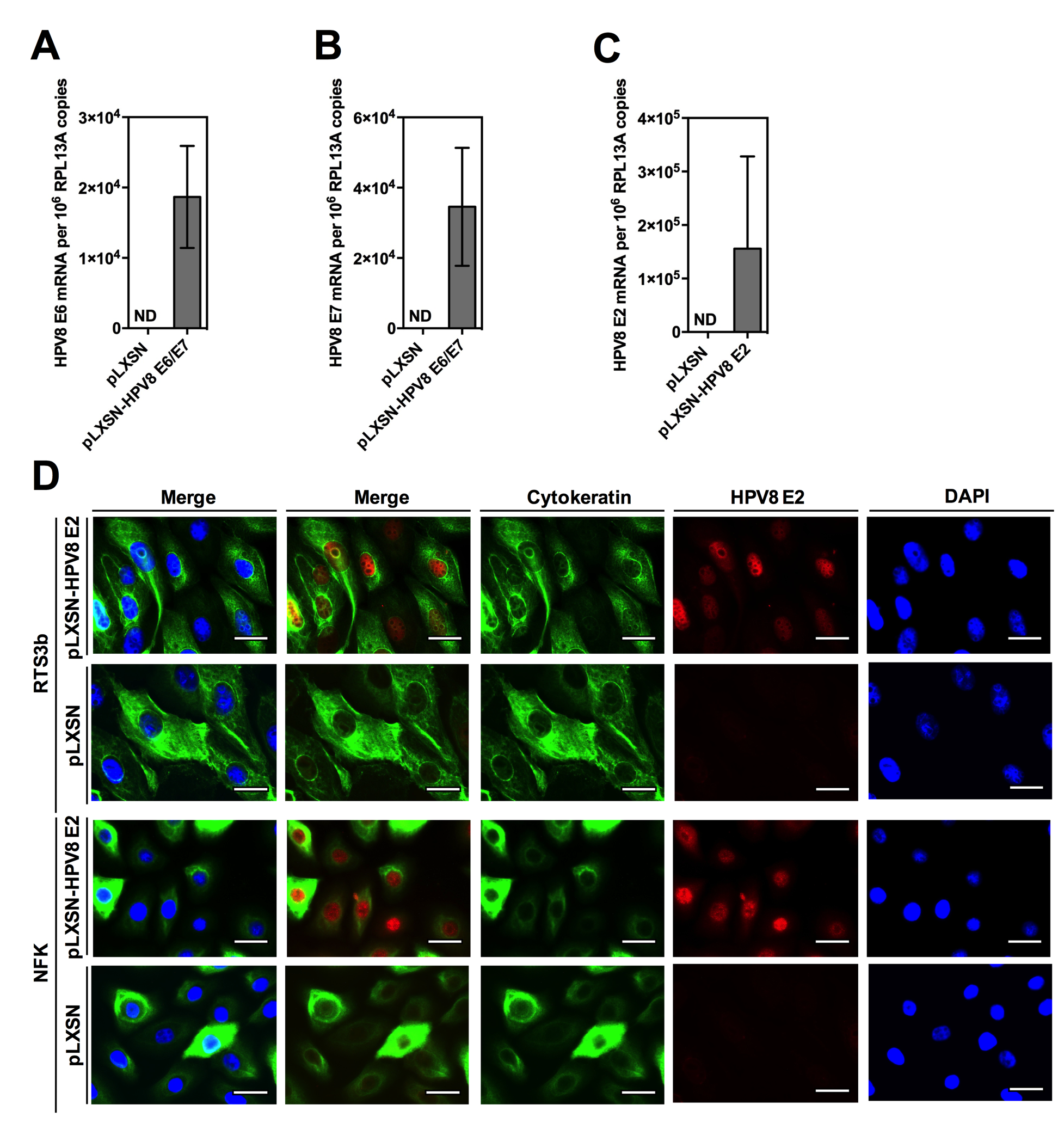


**Supplementary Figure S1.** HPV8 E2, E6 and E7 expression levels in retrovirally infected NFK and RTS3b. NFK stably expressing HPV8 E2 or E6/E7 and control pLXSN cells were analyzed for **(A)** HPV8 E6, **(B)** HPV8 E7 or **(C)** HPV8 E2 by qRT-PCR in relation to RPL13A. Shown are the mean values ± SD from n ≥ 2 independent experiments. **(D)** NFK and RTS3b cells were co-stained for HPV8 E2 (red), cytokeratin (green) and DAPI (blue) by double immunofluorescence. Scale bars 25 µm.
