# Supplementary material for: Chronic Inflammatory Microenvironment in Epidermodysplasia Verruciformis Skin Lesions: Role of the Synergism Between HPV8 E2 and C/EBPβ to Induce Pro-Inflammatory S100A8/A9 Proteins
Source: Front Microbiol. 2018 Mar 7;9:392. doi: 10.3389/fmicb.2018.00392 (PMC5845987; doi:10.3389/fmicb.2018.00392)
Supplement: Supplementary file 2 [file Data_Sheet_2.DOCX]

Supplementary Material

**Chronic inflammatory microenvironment in epidermodysplasia verruciformis skin lesions: role of the synergism between HPV8 E2 and C/EBPβ to induce pro-inflammatory S100A8/A9 proteins**

**Marta Podgórska, Monika Ołdak, Anna Marthaler, Alina Fingerle, Barbara Walch-Rückheim, Stefan Lohse, Cornelia Sigrid Lissi Müller, Thomas Vogt, Mart Ustav, Artur Wnorowski, Magdalena Malejczyk, Sławomir Majewski, Sigrun Smola***

*** Correspondence:** Sigrun Smola: Sigrun.Smola@uks.eu


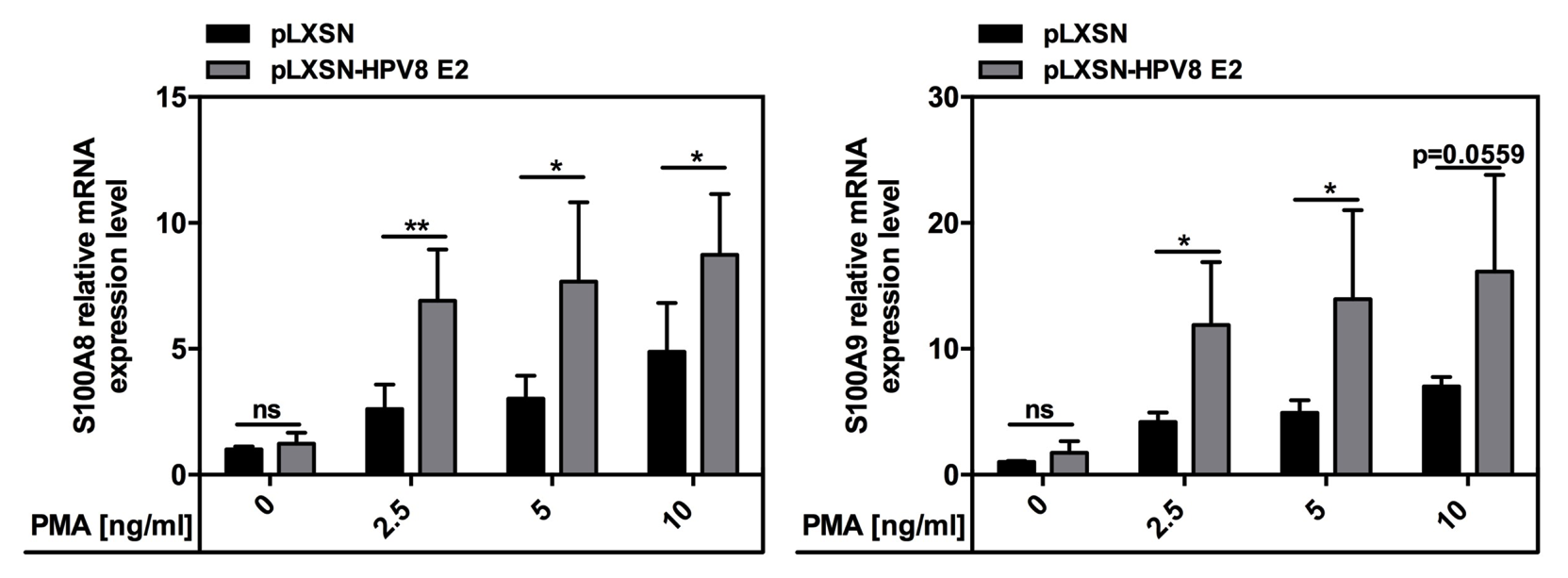


**Supplementary Figure S2.** HPV8 E2 increases PMA-induced expression of S100A8 and S100A9 in NFK cells. NFK cells stably expressing HPV8 E2 or control pLXSN cells were stimulated with PMA or DMSO as a vehicle control and 24 h later S100A8 and S100A9 mRNA levels were measured by qRT-PCR in relation to RPL13A. Shown are the mean values ± SD from n = 2 independent experiments performed in duplicates. ns: not significant, * p < 0.05, ** p < 0.01, unpaired t-test.
