# Supplementary material for: Chronic Inflammatory Microenvironment in Epidermodysplasia Verruciformis Skin Lesions: Role of the Synergism Between HPV8 E2 and C/EBPβ to Induce Pro-Inflammatory S100A8/A9 Proteins
Source: Front Microbiol. 2018 Mar 7;9:392. doi: 10.3389/fmicb.2018.00392 (PMC5845987; doi:10.3389/fmicb.2018.00392)
Supplement: Supplementary file 3 [file Data_Sheet_3.DOCX]

Supplementary Material

**Chronic inflammatory microenvironment in epidermodysplasia verruciformis skin lesions: role of the synergism between HPV8 E2 and C/EBPβ to induce pro-inflammatory S100A8/A9 proteins**

**Marta Podgórska, Monika Ołdak, Anna Marthaler, Alina Fingerle, Barbara Walch-Rückheim, Stefan Lohse, Cornelia Sigrid Lissi Müller, Thomas Vogt, Mart Ustav, Artur Wnorowski, Magdalena Malejczyk, Sławomir Majewski, Sigrun Smola***

*** Correspondence:** Sigrun Smola: Sigrun.Smola@uks.eu


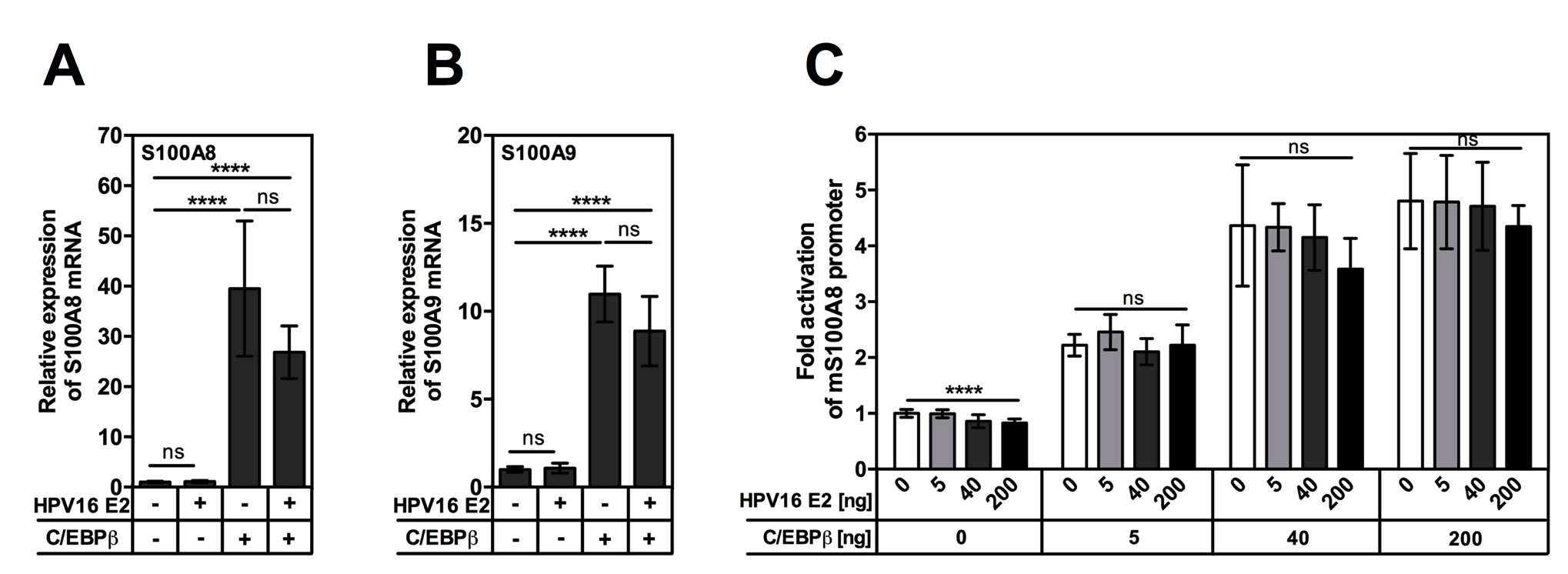


**Supplementary Figure S3.** HPV16 E2 protein does not synergize with C/EBPβ to induce S100A8 and S100A9 expression. 0.4 × 10^6^ RTS3b cells were seeded onto 6-cm dishes, next day co-transfected with 2.64 µg HPV16 E2 and 0.1 µg C/EBPβ expression vectors and 24 h later were analyzed for **(A)** S100A8 and **(B)** S100A9 mRNA expression by qRT-PCR in relation to RPL13A. Shown are the mean values ± SD from n = 3 independent experiments performed in duplicates. **(C)** 0.61 × 10^5^ RTS3b cells were seeded onto 12-well plates and next day co-transfected with 0.2 µg S100A8 reporter vector together with 0.005, 0.04 or 0.2 µg HPV16 E2 and 0.005, 0.04 or 0.2 µg C/EBPβ expression vectors. Total amount of DNA was adjusted up to 0.8 µg with empty pcDNA3.1+ vector. 24 h later the luciferase activity was assayed and normalized to protein concentration. Control transfection was set at 1. Shown are the mean values ± SD from n = 3 independent experiments performed in triplicates. ns: not significant, **** p < 0.0001, unpaired t-test.
