# Supplementary material for: Chronic Inflammatory Microenvironment in Epidermodysplasia Verruciformis Skin Lesions: Role of the Synergism Between HPV8 E2 and C/EBPβ to Induce Pro-Inflammatory S100A8/A9 Proteins
Source: Front Microbiol. 2018 Mar 7;9:392. doi: 10.3389/fmicb.2018.00392 (PMC5845987; doi:10.3389/fmicb.2018.00392)
Supplement: Supplementary file 4 [file Data_Sheet_4.DOCX]

Supplementary Material

**Chronic inflammatory microenvironment in epidermodysplasia verruciformis skin lesions: role of the synergism between HPV8 E2 and C/EBPβ to induce pro-inflammatory S100A8/A9 proteins**

**Marta Podgórska, Monika Ołdak, Anna Marthaler, Alina Fingerle, Barbara Walch-Rückheim, Stefan Lohse, Cornelia Sigrid Lissi Müller, Thomas Vogt, Mart Ustav, Artur Wnorowski, Magdalena Malejczyk, Sławomir Majewski, Sigrun Smola***

*** Correspondence:** Sigrun Smola: Sigrun.Smola@uks.eu


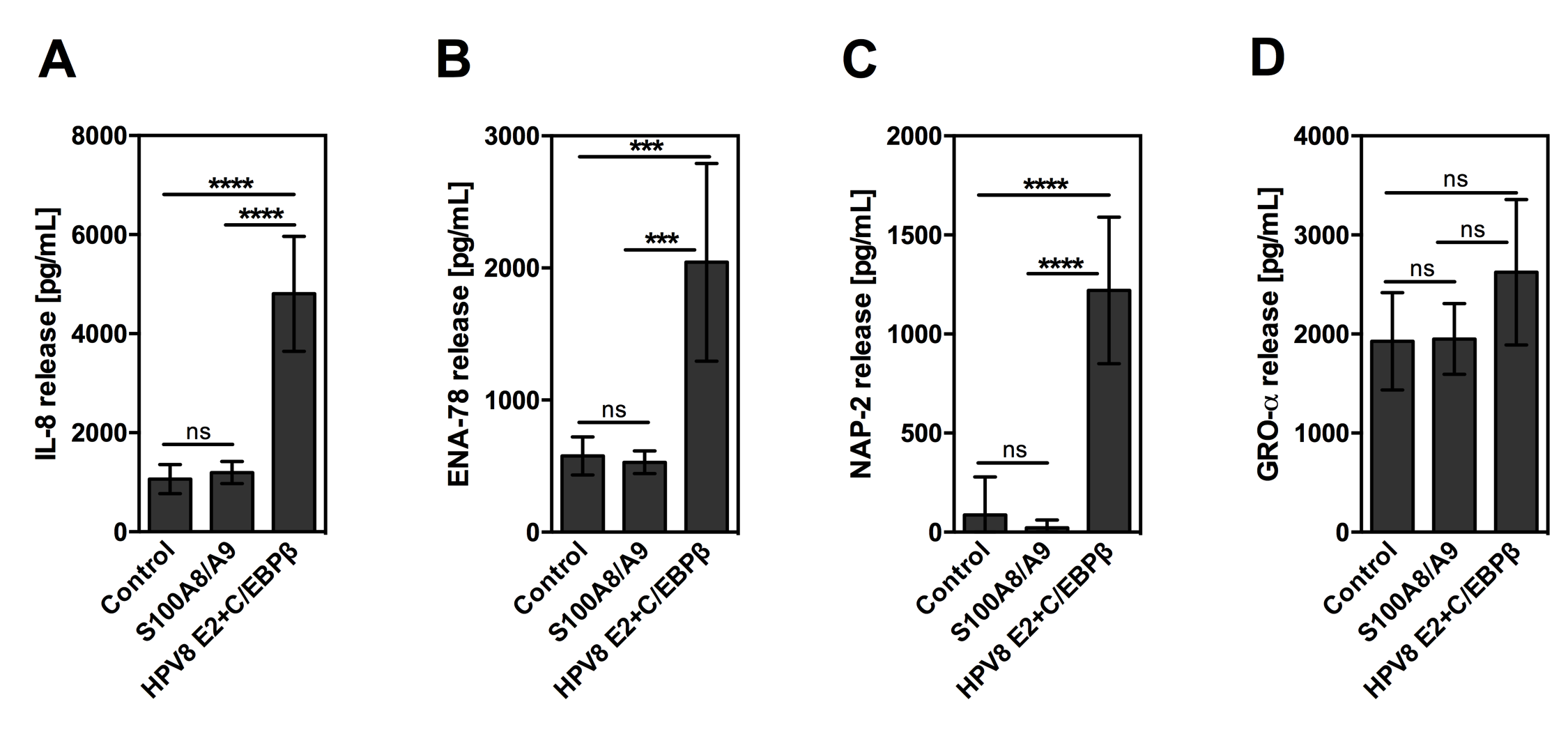


**Supplementary Figure S4.** IL-8, ENA-78, NAP-2 and GRO-α levels in conditioned media of S100A8/A9- or HPV8 E2/C/EBPβ-expressing RTS3b cells. Supernatants used in migration assays were analyzed for **(A)** IL-8, **(B)** ENA-78, **(C)** NAP-2 and **(D)** GRO-α release by ELISA. HPV16 E2 protein does not synergize with C/EBPβ to induce S100A8 and S100A9 expression. Shown are the mean values ± SD from n = 3 independent experiments performed in duplicates. ns: not significant, *** p<0.001, **** p < 0.0001, unpaired t-test.
